# Supplementary material for: Role and mechanism of NCAPD3 in promoting malignant behaviors in gastric cancer
Source: Front Pharmacol. 2024 Apr 22;15:1341039. doi: 10.3389/fphar.2024.1341039 (PMC11070777; doi:10.3389/fphar.2024.1341039)
Supplement: Supplementary file 11 [file DataSheet2.ZIP › GSEA/immunologic/my_analysis.Gsea.1599462003817/GSE16450_CTRL_VS_IFNA_6H_STIM_IMMATURE_NEURON_CELL_LINE_DN.html]

Details for gene set GSE16450\_CTRL\_VS\_IFNA\_6H\_STIM\_IMMATURE\_NEURON\_CELL\_LINE\_DN[GSEA]

|  || Dataset | filtered\_dataset.sample\_info.cls#WT\_versus\_NCAPD3\_MUT |
| Phenotype | sample\_info.cls#WT\_versus\_NCAPD3\_MUT |
| Upregulated in class | NCAPD3\_MUT |
| GeneSet | GSE16450\_CTRL\_VS\_IFNA\_6H\_STIM\_IMMATURE\_NEURON\_CELL\_LINE\_DN |
| Enrichment Score (ES) | -0.4840754 |
| Normalized Enrichment Score (NES) | -2.4764588 |
| Nominal p-value | 0.002570694 |
| FDR q-value | 0.014583043 |
| FWER p-Value | 0.127 |
Table: GSEA Results Summary

  

Fig 1: Enrichment plot: GSE16450\_CTRL\_VS\_IFNA\_6H\_STIM\_IMMATURE\_NEURON\_CELL\_LINE\_DN      
 Profile of the Running ES Score & Positions of GeneSet Members on the Rank Ordered List

  

| SYMBOL | TITLE | RANK IN GENE LIST | RANK METRIC SCORE | RUNNING ES | CORE ENRICHMENT || 1 | 388969 | C2orf68 | 10 | 1.097 | 0.0553 | No |
| 2 | 51512 | GTSE1 | 127 | 0.774 | 0.0156 | No |
| 3 | 85315 | PAQR8 | 351 | 0.567 | -0.1133 | No |
| 4 | 5295 | PIK3R1 | 467 | 0.493 | -0.1683 | No |
| 5 | 11329 | STK38 | 717 | 0.365 | -0.3274 | No |
| 6 | 4209 | MEF2D | 910 | -0.337 | -0.4469 | No |
| 7 | 301 | ANXA1 | 914 | -0.342 | -0.4296 | No |
| 8 | 10123 | ARL4C | 957 | -0.377 | -0.4384 | No |
| 9 | 901 | CCNG2 | 998 | -0.405 | -0.4442 | No |
| 10 | 51646 | YPEL5 | 1014 | -0.413 | -0.4315 | No |
| 11 | 10938 | EHD1 | 1019 | -0.419 | -0.4106 | No |
| 12 | 26053 | AUTS2 | 1113 | -0.485 | -0.4501 | No |
| 13 | 388403 | YPEL2 | 1161 | -0.527 | -0.4541 | Yes |
| 14 | 153222 | CREBRF | 1187 | -0.559 | -0.4403 | Yes |
| 15 | 9665 | KIAA0430 | 1192 | -0.566 | -0.4109 | Yes |
| 16 | 472 | ATM | 1193 | -0.568 | -0.3785 | Yes |
| 17 | 9975 | NR1D2 | 1230 | -0.605 | -0.3701 | Yes |
| 18 | 57494 | RIMKLB | 1233 | -0.610 | -0.3367 | Yes |
| 19 | 54491 | FAM105A | 1241 | -0.619 | -0.3065 | Yes |
| 20 | 23508 | TTC9 | 1271 | -0.660 | -0.2899 | Yes |
| 21 | 57561 | ARRDC3 | 1324 | -0.744 | -0.2850 | Yes |
| 22 | 5997 | RGS2 | 1336 | -0.770 | -0.2491 | Yes |
| 23 | 113791 | PIK3IP1 | 1345 | -0.787 | -0.2100 | Yes |
| 24 | 10397 | NDRG1 | 1380 | -0.925 | -0.1819 | Yes |
| 25 | 26959 | HBP1 | 1395 | -1.073 | -0.1309 | Yes |
| 26 | 493 | ATP2B4 | 1401 | -1.166 | -0.0680 | Yes |
| 27 | 51274 | KLF3 | 1406 | -1.296 | 0.0029 | Yes |
Table: GSEA details [plain text format]

  

Fig 2: GSE16450\_CTRL\_VS\_IFNA\_6H\_STIM\_IMMATURE\_NEURON\_CELL\_LINE\_DN      
 Blue-Pink O' Gram in the Space of the Analyzed GeneSet

  

Fig 3: GSE16450\_CTRL\_VS\_IFNA\_6H\_STIM\_IMMATURE\_NEURON\_CELL\_LINE\_DN: Random ES distribution      
 Gene set null distribution of ES for **GSE16450\_CTRL\_VS\_IFNA\_6H\_STIM\_IMMATURE\_NEURON\_CELL\_LINE\_DN**
